# Pharmacological Inhibition of Gal-3 in Mesenchymal Stem Cells Enhances Their Capacity to Promote Alternative Activation of Macrophages in Dextran Sulphate Sodium-Induced Colitis

**DOI:** 10.1155/2016/2640746

**Published:** 2016-01-04

**Authors:** Bojana Simovic Markovic, Aleksandar Nikolic, Marina Gazdic, Jasmin Nurkovic, Irena Djordjevic, Nebojsa Arsenijevic, Miodrag Stojkovic, Miodrag L. Lukic, Vladislav Volarevic

**Affiliations:** ^1^Center for Molecular Medicine and Stem Cell Research, Faculty of Medical Sciences, University of Kragujevac, 69 Svetozar Markovic Street, 34000 Kragujevac, Serbia; ^2^Stem Cell Laboratory, Department of Biomedical Sciences, State University of Novi Pazar, Nn Vuk Karadzic Street, 36300 Novi Pazar, Serbia; ^3^Spebo Medical, 16 Norvezanska Street, 16000 Leskovac, Serbia

## Abstract

Transplantation of mesenchymal stem cells (MSCs) reduces the severity of dextran sulphate sodium- (DSS-) induced colitis. MSCs are able to secrete Galectin-3 (Gal-3), a protein known to affect proliferation, adhesion, and migration of immune cells. We investigate whether newly synthetized inhibitor of Gal-3 (*Davanat*) will affect production of Gal-3 in MSCs and enhance their potential to attenuate DSS-induced colitis. Pharmacological inhibition of Gal-3 in MSCs enhances their capacity to promote alternative activation of peritoneal macrophages *in vitro* and *in vivo*. Injection of MSCs cultured in the presence of Davanat increased concentration of IL-10 in sera of DSS-treated animals and markedly enhanced presence of alternatively activated and IL-10 producing macrophages in the colons of DSS-treated mice. Pharmacological inhibition of Gal-3 in MSCs significantly attenuates concentration of Gal-3 in sera of DSS-treated animals, indicating that MSCs produce Gal-3 in this disease. In conclusion, our findings indicate that Davanat could be used for improvement of MSC-mediated polarization towards immunosuppressive M2 phenotype of macrophages.

## 1. Introduction

Ulcerative colitis (UC) and Crohn's disease (CD) are the two major forms of inflammatory bowel disease (IBD) and are characterized by an abnormal cell influx to the intestinal tissues and massive release of proinflammatory mediators [1]. One of the most common IBD-related animal models is the* Dextran Sulphate Sodium*- (*DSS*-) induced colitis, originally reported by Okayasu et al. [2]. The clinical features of the DSS-induced colitis are similar to human colitis and include weight loss, loose stool/diarrhea, and occult and gross rectal bleeding. DSS has a toxic effect on epithelial cells, resulting in invasion of intestinal bacteria into subepithelial tissue. Dendritic cells (DCs) and macrophages capture bacteria that have passed through DSS-injured colonic epithelium, and through activation of Toll-like receptors (TLRs), release proinflammatory cytokines (TNF-*α*, IL-12) and chemokines (macrophage inflammatory protein- (MIP-) 1*α*, monocyte chemotactic protein- (MCP-) 1, and keratinocyte-derived chemokine (CXCL1/KC), CCL11) which induce migration of inflammatory cells in the colon [3, 4].

Mesenchymal stem cells (MSCs) are adult, multipotent cells that can be found in almost all postnatal tissues [5, 6]. MSCs can alter immune response and regulate the proliferation, activation, and effector function of T lymphocytes, professional antigen presenting cells (DCs, macrophages, B lymphocytes), and NK cells, through cell-to-cell contact or through the production of soluble factors [7]. Due to their immunomodulatory properties, much interest has been focused in MSC-based therapy of inflammatory disorders, including IBD [8–10].

Recently, transplantation of MSCs has been found to reduce the severity of DSS-induced colitis [11–14]. Liu et al. [15] found that MSCs significantly alleviated the DSS-induced colitis, and the major sources for TGF-*β*1 were macrophages that were recruited by MSCs. Specific ablation of macrophages completely abolished the anti-inflammatory effects of MSCs [15].

Sioud et al. [16] showed that MSCs were able to secrete Galectin-3 (Gal-3), a protein known to affect proliferation, adhesion, and migration of immune cells. Because Gal-3 is widely expressed in immune cells (neutrophils, eosinophils, basophils, mast cells, DCs, monocytes, and macrophages, as well as in NK cells) [17], which are involved in pathogenesis of DSS-induced colitis, we investigated the role of Gal-3 produced by MSCs in this experimental model.

## 2. Materials and Methods

### 2.1. Cells

Murine MSCs isolated from bone marrow of C57BL/6 mice were purchased from Gibco (catalog number S10502-01). The cells were cultured in* Dulbecco's Modified Eagle Medium* (DMEM) containing 10% heat-inactivated fetal bovine serum (FBS), 100 IU/mL penicillin G, and 100 *μ*g/mL streptomycin (Sigma-Aldrich Chemical, Munich, Germany), at 37°C in a 5% CO_2_ incubator. MSCs in passage 6 were used throughout these experiments.

### 2.2. Animals

We used 6–8-week-old male wild type (WT) C57BL/6 mice for induction of DSS colitis. Male, 6–8-week-old, Gal-3^−/−^ C57BL/6 mice (provided by Dr. Daniel Hsu, University of California, Sacramento, CA) were used in the coculture experiments. Targeted disruption of mouse Gal-3 gene was performed in C57BL/6 embryonic stem cells and mice homozygous for disrupted gene were obtained [18]. Mice were maintained in animal breeding facilities of Faculty of Medical Sciences, University of Kragujevac, Serbia. All animals received human care and all experiments were approved by and conducted in accordance with the Guidelines of the Animal Ethics Committee of the Faculty of Medical Sciences, University of Kragujevac, Serbia. Mice were housed in a temperature-controlled environment with a 12-hour light-dark cycle and were administered standard laboratory chow and water* ad libitum*.

### 2.3. Experimental Design

Experimental animals were divided into 4 groups: (1) wild type (WT); (2) WT + DSS (Dextran Sulphate Sodium); (3) WT + DSS + MSCs (mesenchymal stem cells); and (4) WT + DSS + MSCs + Davanat (Gal-3 inhibitor). Each group had 10 animals. To complete this study, 80 WT and 10 Gal-3^−/−^ C57BL/6 animals were used.

### 2.4. Induction of Acute Colitis

Colitis was induced in C57BL/6 with 3% w/v DSS (molecular weight 40 kDa; TdB Consultancy, Uppsala, Sweden) dissolved in drinking water given* ad libitum* (days 1–7) as previously described [2]. Control mice were given DSS-free water.

### 2.5. Administration of MSCs

On day 0, 12 h after DSS administration, mice were injected intraperitoneally (ip) with 0.5 × 10^6^ MSCs diluted in 200 mL PBS or a vehicle control (PBS alone) [19].

### 2.6. Pharmacological Inhibition of Gal-3 in MSCs

In order to inhibit production of Gal-3 in MSCs, MSCs were treated with inhibitor of Gal-3 (Davanat, 15 *μ*g/mL, kindly provided by Professor Klyosov and Professor Traber from Galectin Therapeutics Inc., Newton, MA) and MSCs + Davanat were administered on day 0 (the 1st day of DSS administration), intraperitoneally, according to previously published protocol [20].

### 2.7. Assessment of the Severity of Colitis

*Disease Activity Index* (DAI) was used to assess the clinical signs of colitis (Table 1). Body weight was measured daily and compared with the body weight measured on day 0 (the 1st day of DSS administration). The obtained results were presented as ±% body weight loss. The analysis of stool consistency and Hemoccult (Beckman Coulter) fecal occult blood test were performed daily [21].

### 2.8. Histology

For histological analysis, colons were removed from euthanized mice, rinsed with phosphate buffer solution (PBS), and cut longitudinally before being rolled into “Swiss roll,” as previously described [22]. Swiss-rolled colons were fixed in formalin and embedded in paraffin and 5 *μ*m sections were stained with hematoxylin and eosin (H&E) and examined in a blinded manner by pathologist. Sections were analyzed for damage of epithelium including damage of crypts, submucosal edema, hemorrhage, and infiltration of immune cells. The histology scores for each mouse were calculated as the sum of “infiltration” and “damage of epithelium” subscores, as previously described (Table 2) [23].

### 2.9. Measurements of Cytokines in Serum

We used the commercial ELISA sets (R&D Systems, Minneapolis, MN) to measure the concentration of selected cytokines (Gal-3, TNF-*α*, IL-1*β*, IL-10, and TGF-*β*) according to the manufacturer's instructions [24]. Briefly, blood sample was collected from abdominal aorta during euthanasia procedure. Serum was separated by centrifugation and stored at −80°C. Gal-3, TNF-*α*, IL-1*β*, IL-10, and TGF-*β* serum levels were measured by enzyme-linked immunosorbent assay (ELISA).

### 2.10. Isolation of Immune Cells from Lamina Propria and Flow Cytometry Analysis

Isolation of immune cells from* lamina propria* was conducted as previously described [25]. Briefly, each colon was dissected away from cecum. The colons were cut in 3 cm long pieces and then cut longitudinally, so that 3 × 3 cm flaps of colonic tissue were made. The flaps were placed in a 50 mL conical tube and washed 3 to 5 times with 30 mL cold HBSS, calcium- and magnesium-free. After decanting the supernatant, the pieces were incubated in 20 mL HBSS/EDTA for 30 min in a 37°C water bath. Each tube was shaken regularly during the incubation to ensure that epithelial cells are disrupted from the mucosa. The pieces were sediment and supernatants were decanted. The remaining EDTA were washed out with 40 mL HBSS, calcium- and magnesium-free. The fragments of colonic tissue were placed in a 10 cm petri dish and cut into smaller pieces with a razor blade or scalpel. The pieces were aspirated with a pipette, transferred to a new 50 mL conical tube, and filled to 20 mL with DMEM supplemented with 10% fetal bovine serum (FBS). Then, 1 mL of 4000 Mandl units (3 × 10^6^ Wünsch units)/mL collagenase D and 200 *μ*L of 1 mg/mL DNase were added to the tube and incubated for 1 h in a 37°C water bath. The supernatants were filtered through a 100 *μ*m nylon cell strainer into a clean 50 mL conical tube. A cold HBSS, calcium- and magnesium-free, was added to 50 mL. Cells were pelleted by centrifuging for 10 min at 450 ×g, at 4°C. The pellet was disrupted and cells were resuspended in 50 mL HBSS, calcium- and magnesium-free, and filtered through a 40 *μ*m nylon cell strainer into a clean 50 mL conical tube. Cells were again pelleted by centrifuging for 10 min at 450 ×g, 4°C. The pellet was disrupted and cells were resuspended in 20 mL of 30% Percoll. Then, the cell suspension was carefully layered over 25 mL of 70% Percoll in a 50 mL conical tube and centrifuged for 20 min at 1100 ×g, room temperature, with as low an acceleration rate as possible and with the brake off. Clumping of cells was prevented by the addition of 1 mM EDTA to the solution. Epithelial cells float on the 30% Percoll layer, while immune cells were found between the 30% and 70% layer. Debris and dead cells were pelleted at the bottom of the conical tube.

Flow cytometry followed routine procedures by using 1 × 10^6^ cells per sample and were incubated with antimouse F4/80, antimouse CD206, antimouse Fc*ε*RI, antimouse CD117, antimouse CD11c, antimouse CD11b, antimouse CD80, antimouse NK1.1, and antimouse CD3 conjugated with fluorescein isothiocyanate (FITC; BD Biosciences, Franklin Lakes, NJ), phycoerythrin (PE; BD Biosciences), peridinin chlorophyll protein (PerCP; BD Biosciences), or allophycocyanin (APC; BD Biosciences). For the intracellular staining, cells were previously stimulated with phorbol myristate acetate (PMA) and ionomycin for 4 h at 37°C. Following extracellular staining, cells were fixed, permeabilized, and stained for TNF-*α*, IL-10, IL-12, and IL-1*β* by using conjugated antimouse antibodies. Flow cytometric analysis was conducted on a BD Biosciences FACSCalibur and analyzed by using the Flowing software analysis program.

### 2.11. Isolation and* In Vitro* Coculture of Peritoneal Macrophages

Macrophages were isolated from peritoneal cavity of untreated, healthy Gal-3^−/−^ mice. Mice were injected with 5 mL of PBS ip and, after shaking, peritoneal lavage was performed. Macrophages were collected from the peritoneal cavity of mice under sterile conditions and cultured in complete DMEM supplemented with 10% FBS at 37°C in a 5% CO_2_ incubator. Isolated macrophages were plated at a density of 10^6^ cells/well and cocultured with MSCs and MSCs + Davanat cells for 24 hours [26]. The levels of IL-10 and TGF-*β* were determined in cell culture supernatants by ELISA sets, according to manufacturer's recommendations. Phenotype of macrophages was determined by flow cytometry, as described above.

### 2.12. Statistics

Data were expressed as the mean ± SEM for each group. We tested for normality using Shapiro-Wilk's test and for homogeneity of variances using Levine's test. A paired samples *t*-test was used to compare two matched groups. Independent samples Student's *t*-test was otherwise used to compare two groups with Gaussian distribution. Fisher's exact test was used to assess survival differences between groups. Statistical analyses were performed using SPSS 19.0 for Windows software (SPSS, Inc., Chicago, IL). All reported *P* values were 2-sided and *P* < 0.05 was considered statistically significant and highly significantly different when *P* < 0.01.

## 3. Results

### 3.1. Pharmacological Inhibition of Gal-3 in MSCs Significantly Attenuates Concentration of Gal-3 in Sera of DSS-Treated Mice

**Figure 1:**
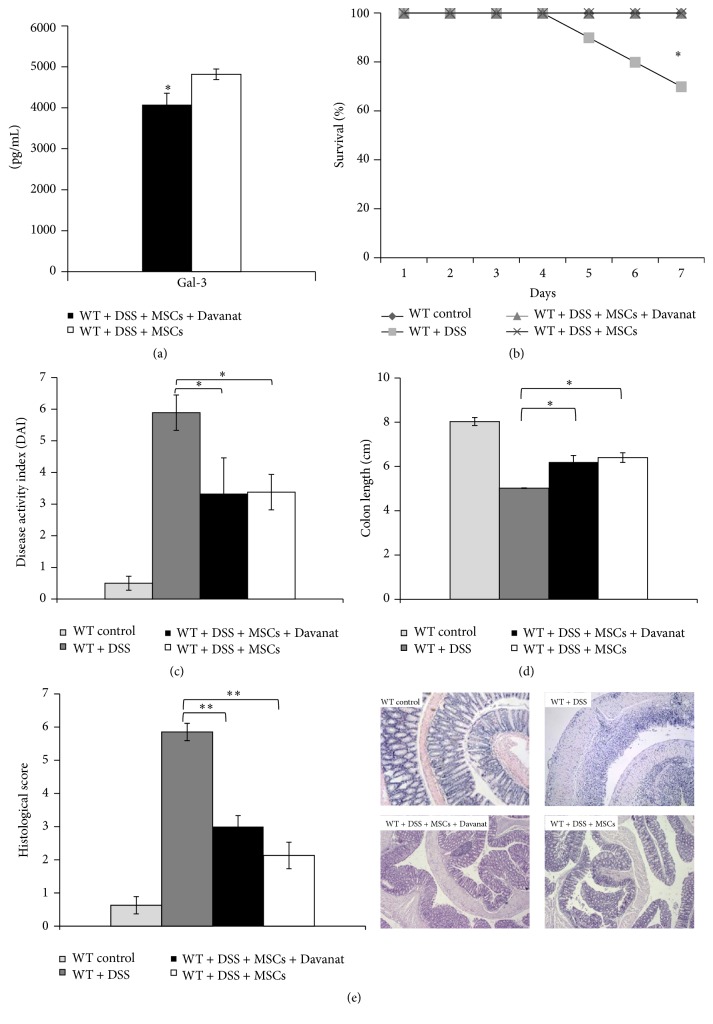
MSCs attenuate DSS-induced colitis. Water with 3% DSS was given to mice for 7 days; regular drinking water was fed to control mice. The concentration of Gal-3 in sera of MSCs groups (a). Survival rate of mice with colitis (b). Disease Activity Index (DAI) scored at day 7 using the following parameters: weight loss, stool consistency, and rectal bleeding (c). After DSS treatment length of the entire colon was measured (d). Histological examination was performed with hematoxylin and eosin staining (e). H&E staining images of representative colon tissues are shown at the same magnifications (100x) (e). Data presented as means ± SEM; *n* = 10 mice per experimental groups. ^*∗*^ *P* < 0.05, ^*∗∗*^ *P* < 0.01.

The concentration of Gal-3 in sera of DSS-treated mice that received MSCs correlates with pharmacological inhibition of this molecule in MSCs. As it is shown in Figure 1(a), pharmacological inhibition of Gal-3 in MSCs significantly attenuates concentration of Gal-3 in sera of DSS-treated animals, indicating that MSCs produce Gal-3 in this disease (*P* < 0.05; Student's* t-*test).

Inhibition of Gal-3 in MSCs did not alter their potential to prevent the development of DSS-induced colitis, according to survival rate (Figure 1(b)) (*P* < 0.05; Fisher's exact test), clinical parameters (Figure 1(c)), and colon length (Figure 1(d)) (*P* < 0.05; Student's* t*-test). All DSS-treated WT mice developed severe colitis with similar clinical symptoms: diarrhea, rectal bleeding, and weight loss. The presence of blood in the feces was detected one to two days after the start of DSS treatment, whereas gross bleeding and diarrhea were initially observed from day 4. Significant body weight loss (>5%) became prominent after four days of DSS treatment. Compared to DSS-only-treated animals, DSS-treated mice that received MSCs + Davanat or MSCs did not develop diarrhea, gross rectal bleeding, and significant body weight loss till the end of the experiment. These observations were also confirmed by the histological analysis (Figure 1(e)) (*P* < 0.01; Student's* t*-test). The DSS-treated group clearly exhibited a severe mucosal inflammatory cell infiltrate and disruption of crypt architecture (epithelial ulcerations and loss of goblet cells), whereas DSS-induced lesions were prevented in both MSCs + Davanat- and MSCs-only-treated animals (Figure 1(e), lower panels).

### 3.2. Pharmacological Inhibition of Gal-3 in MSCs Results in Increased Concentration of IL-10 in Sera of DSS-Treated Animals

**Figure 2:**
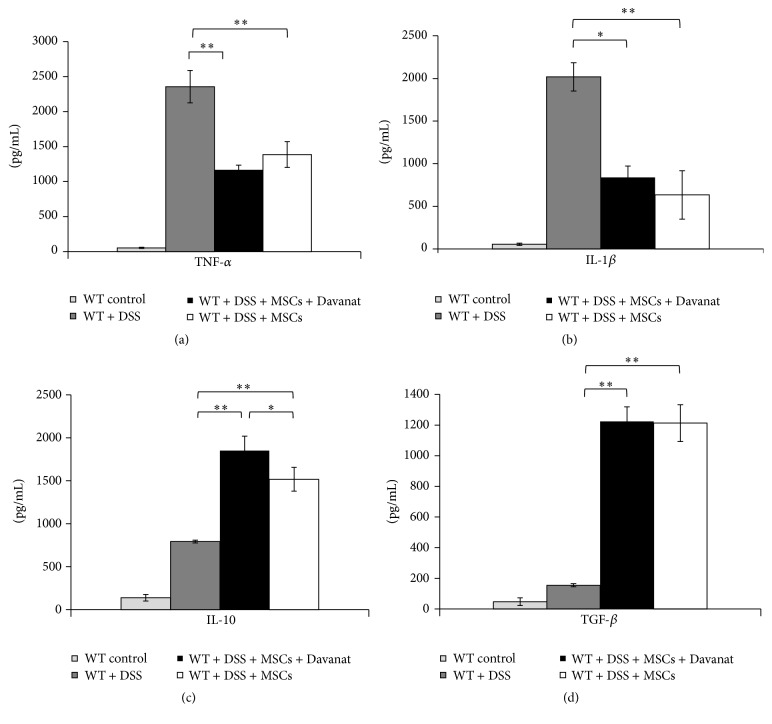
Pharmacological inhibition of Gal-3 in MSCs results in increased concentration of IL-10 in sera of DSS-treated animals. Levels of proinflammatory cytokines in the sera are shown: (a) TNF-*α* and (b) IL-1*β*. Levels of anti-inflammatory cytokines in the sera are shown: (c) IL-10 and (d) TGF-*β*. Values are mean ± SEM (*n* = 10 per group). ^*∗*^ *P* < 0.05, ^*∗∗*^ *P* < 0.01.

In effort to investigate the effect of Gal-3 on immunomodulatory characteristics of MSCs in DSS-induced colitis, we analyzed concentration of cytokines in sera. There were significantly lower levels of inflammatory TNF-*α* and IL-1*β* and significantly higher levels of anti-inflammatory IL-10 and TGF-*β* in sera of DSS-treated mice that received MSCs + Davanat or MSCs only when compared to DSS-only-treated animals (Figure 2) (*P* < 0.01; Student's* t-*test). Importantly, the concentration of IL-10 was significantly higher (Figure 2(c)) (*P* < 0.05; Student's* t-*test) in sera of MSCs + Davanat-treated mice when compared to concentration of this cytokine in MSCs-only-treated mice with DSS-induced colitis.

### 3.3. Pharmacological Inhibition of Gal-3 in MSCs Results in Markedly Enhanced Presence of Alternatively Activated and IL-10 Producing Macrophages in the Colons of DSS-Treated Mice

**Figure 3:**
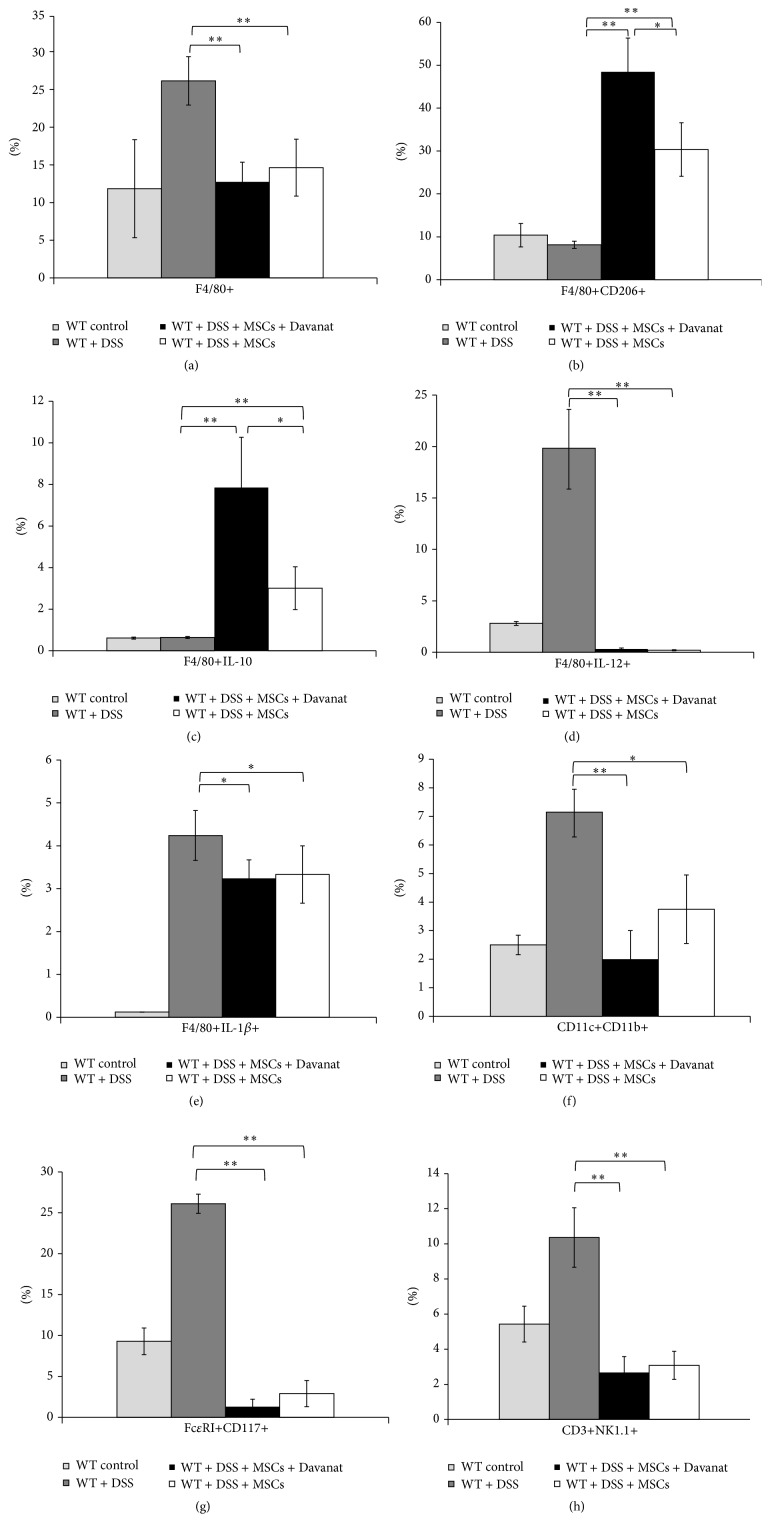
Pharmacological inhibition of Gal-3 in MSCs favored alternative activation of macrophages in the colon tissues of DSS-treated mice. The percentage of F4/80+ macrophages in colon tissue (a). Significant decrease in percentage of F4/80+CD206+ and F4/80+ IL-10+ macrophages in MSCs-treated mice (white bars), when compared to MSCs + Davanat-treated mice (black bars), after 7 days on DSS treatment (b and c). The percentage of IL-12- and IL-1*β*-producing F4/80+ macrophages (d and e). The percentages of inflammatory dendritic cells (f) and Fc*ε*RI+CD117+ mast cells (g) as well as CD3+NK1.1+NKT cells (h) are shown. Values are mean ± SEM (*n* = 10 per group). ^*∗*^ *P* < 0.05, ^*∗∗*^ *P* < 0.01.

Injection of MSCs resulted in significantly lower number of F4/80+ macrophages, Fc*ε*RI+CD117+ mast cells, inflammatory CD11c+CD11b+DCs, and CD3+NK1.1+NKT cells in colons of DSS-treated mice (Figure 3). Among all these cells, pharmacological inhibition of Gal-3 in MSCs affected only phenotype and cytokine production of macrophages (Figures 3(b) and 3(c)) in colons of DSS-treated animals. Although there was not any difference in the percentage of IL-12 and IL-1*β* producing F4/80+ colonic macrophages (Figures 3(d) and 3(e)) between MSCs and MSCs + Davanat groups, pharmacological inhibition of Gal-3 in MSCs results in markedly enhanced presence of alternatively activated and IL-10 producing macrophages in the colons of DSS-treated mice. The percentage of F4/80+CD206+ alternatively activated macrophages as well as macrophages that produced IL-10 was significantly higher in mice that received MSCs in which production of Gal-3 was inhibited (Figures 3(b) and 3(c)) (*P* < 0.05; Student's* t-*test) when compared with other experimental groups.

### 3.4. Pharmacological Inhibition of Gal-3 in MSCs Enhances Their Capacity to Promote Alternative Activation of Peritoneal Macrophages

**Figure 4:**
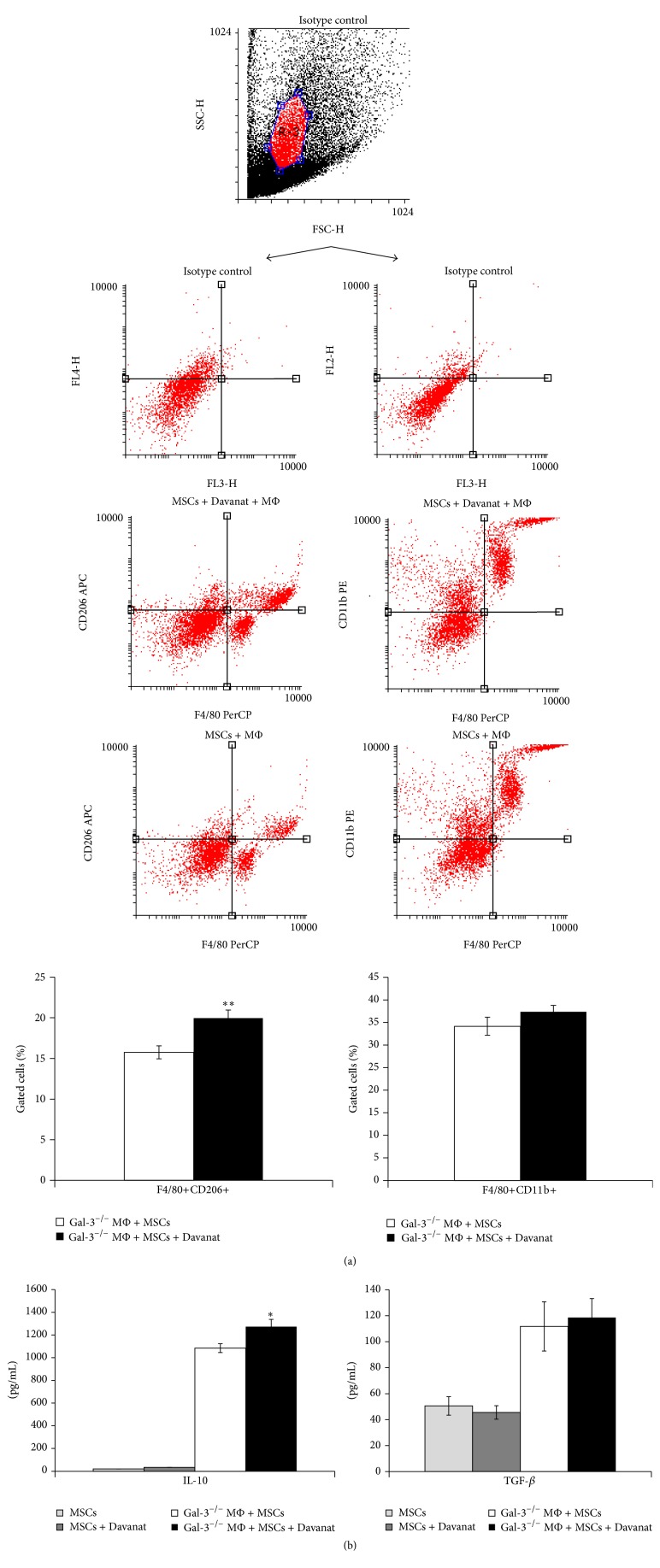
Pharmacological inhibition of Gal-3 in MSCs enhances their capacity to promote alternative activation of peritoneal macrophages. The significant increase in percentage of F4/80+CD206+ macrophages in MSCs + Davanat-treated Gal-3^−/−^ macrophages (black bars), when compared to only MSCs-treated Gal-3^−/−^ macrophages (white bars) (a). The percentage of F4/80+CD11b+ macrophages is shown (a). Representative flow cytometry dot plots are shown. The level of IL-10 and TGF-*β* in supernatants (b). Values are mean ± SEM (*n* = 10 per group). ^*∗*^ *P* < 0.05, ^*∗∗*^ *P* < 0.01.

In order to elucidate the role of Gal-3 produced by MSCs for alternative activation of macrophages, Gal-3^−/−^ macrophages were cocultured with MSCs or MSCs + Davanat cells. Pharmacological inhibition of Gal-3 in MSCs enhances their capacity to promote alternative activation of peritoneal macrophages. There was significantly higher percentage of F4/80+CD206+ alternatively activated macrophages in population of peritoneal macrophages cocultured with MSCs + Davanat cells (Figure 4(a)) (*P* < 0.01; Student's* t-*test), while there was no significant difference in the percentage of inflammatory F4/80+CD11b+ macrophages between experimental groups (Figure 4(a)). In line with these findings, there was a significantly higher level of IL-10 in supernatants of macrophages cocultured with MSCs + Davanat cells (Figure 4(b)) (*P* < 0.05; Student's* t*-test).

## 4. Discussion

The immunoregulatory activity of MSCs offers a novel strategy in the design of therapeutic protocols aimed at suppressing pathologic immune responses responsible for the development of IBD. In this context, it has been shown that MSCs can exert their inhibitory effect not only on cells of adaptive immune response, but also on cells of the innate immunity, including DC, NK cells, and macrophages [7].

Macrophages have been identified as one of the most important cells for the induction of acute human colitis and DSS-induced colitis [27, 28]. Uptake of DSS by macrophages activates the Nlrp3 inflammasome resulting in increased production of inflammatory cytokines IL-1*β* and IL-18. In addition, production of nitric oxide (NO) and expression of inducible nitric oxide synthase (iNOS) in macrophages exacerbate DSS-induced colitis [29].

Recently, Liu and coworkers suggest that MSCs transplantation may recruit macrophages to produce anti-inflammatory cytokines, which attenuate colitis [15]. In line with these findings, we found that MSCs significantly ameliorated the clinical and histopathological severity of DSS-induced colitis (Figure 1) that correlated with increased serum levels of IL-10 (Figure 2) and increased percentage of F4/80+CD206+ alternatively activated macrophages in colon (Figure 3).

A limited number of studies have been performed to investigate a potential of MSCs to educate macrophages to adapt an anti-inflammatory/immune-suppressive phenotype: to express higher levels of CD206 (marker for alternatively activated macrophages) and to increase production of anti-inflammatory cytokine IL-10. Kim and Hematti [30] were first to report that human bone marrow-derived MSC could promote the generation of alternatively activated macrophages.

In another study, Cutler and coworkers showed that monocytes isolated from peripheral blood displayed increased expression of CD206, lower levels of surface HLA-DR, and reduced capability of stimulating alloreactive T-cell response, after coculturing with umbilical cord-derived MSC [31]. Zhang et al. [32] showed that human gingiva-derived MSCs could induce polarization of M2 macrophages* in vitro* and confirmed this phenomenon* in vivo*. They reported that repeatedly infused human gingiva-derived MSCs could home to the wound site in close proximity with host macrophages and promote their polarization towards M2 phenotype [32].

Concerning the mechanisms underlying the M2 polarizing effect exerted by MSCs on macrophages, an essential role of different soluble factors has been demonstrated. By the use of specific neutralizing antibodies, Zhang and colleagues [32] showed an involvement of IL-6 and granulocyte-macrophage-CSF while Cutler et al. [31] reported the importance of PGE2 for the induction of M2 phenotype by MSC.

Herewith, we showed that Davanat-mediated pharmacological inhibition of Gal-3 in MSCs resulted in enhanced presence of F4/80+CD206+ alternatively activated and IL-10 producing macrophages in colon of DSS-treated animals (Figure 3) and increased serum levels of IL-10 (Figure 2) indicating the importance of Gal-3 produced by MSCs, for macrophage polarization towards M2 phenotype. In addition, we showed that pharmacological inhibition of Gal-3 in MSCs enhances capacity of MSCs to promote M2 polarization of macrophages and IL-10 production* in vitro* (Figure 4).

It is well known that Gal-3 plays important role in macrophage polarization and function [33–36]. In animal model of type 1 diabetes, macrophages of Gal-3 deficient mice produce less TNF-alpha and nitric oxide (NO) and are less effective in intracellular and extracellular killing compared with WT mice [37]. By using an animal model of immune mediated acute hepatitis [36], we showed that both genetic deletion and TD139-induced pharmacological inhibition of Gal-3 resulted in an increased number of IL-10-producing alternatively activated, M2-polarized macrophages in the livers.

Traber and coworkers demonstrated that Davanat significantly reduced expression of Gal-3 in portal and septal macrophages resulting in attenuated fibrosis in thioacetamide-induced liver disease [38]. Also, pharmacological inhibitor of Gal-3 managed to ameliorate hepatocellular damage, inflammation, and fibrosis in a mouse model of nonalcoholic fatty liver disease [39] and these effects are associated with a reduction of Gal-3 expression on liver macrophages.

## 5. Conclusion

In conclusion, Davanat-induced inhibition of Gal-3 did not significantly affect potential of MSCs to attenuate colitis but managed to enhance production of anti-inflammatory cytokine IL-10 in colonic macrophages and to promote their polarization towards immunosuppressive M2 phenotype. Our findings indicate that Gal-3 target drugs could be used for improvement of MSCs-mediated suppression of macrophages.

## Figures and Tables

**Table 1:** Criteria for scoring the Disease Activity Index of IBD (DAI)^*∗*^.

Score	Weight loss	Stool consistency	Visible blood in feces
0	No weight loss	Normal	No bleeding
1	1–5%		
2	6–10%	Loose	Slight bleeding
3	11–15%		
4	>15%	Diarrhea	Gross bleeding

^*∗*^DAI value is calculated as the sum of scores of weight loss, stool consistency, and blood in feces.

**Table 2:** Histological scoring^*∗*^.

Score	Infiltration	Damage of epithelium
0	No infiltration	Normal morphology

1	Infiltration around crypt basis	Loss of goblet cells

2	Infiltration reaching the lamina muscularis mucosae	Loss of goblet cells in large areas

3	Extensive infiltration reaching the lamina muscularis mucosae associated with mucosa thickening and oedema	Loss of crypts

4	Infiltration of the lamina submucosa	Loss of crypts in large areas

^*∗*^Scores were calculated by adding the score for two parameters, giving a maximum score of 8.
